# Deciphering the needs of patients with hereditary breast and ovarian Cancer in the Process of Genetic Counseling to Inform the Development of a Mobile Support App: a qualitative study in Germany

**DOI:** 10.1007/s12687-024-00727-6

**Published:** 2024-08-19

**Authors:** Nils Ammon, Chiara Reichert, Thomas Kupka, Steffen Oeltze-Jafra, Anke Katharina Bergmann, Brigitte Schlegelberger, Dominik Wolff, Beate Vajen

**Affiliations:** 1 Department of Human Genetics, Hannover Medical School, Hannover, Germany; 2 Peter L. Reichertz Institute for Medical Informatics of TU Braunschweig and Hannover Medical School, Hannover, Germany

**Keywords:** Genetic counseling, Mobile app, Web application, Digital tool, Patient-centered support tool, HBOC

## Abstract

Patients with hereditary breast and ovarian cancer (HBOC) are not only concerned about their own health but also about that of their children, grandchildren, and other relatives. Therefore, they have specific needs for information and support. During genetic counseling guidance is provided to HBOC patients and other individuals who may be at risk for familial cancer. The purpose of the study was to identify the needs of HBOC patients during the genetic counseling process that could be addressed by digital solutions. Nine semi-structured qualitative interviews were conducted. Overall, the patients appreciated the personal contact with human geneticists as an especially positive factor in the genetic counseling process. However, patients noted the following needs (1) support in the time following genetic counseling, (2) support before genetic counseling by collecting own and familial medical information, (3) Need for contact options to support services, (4) Need for patient-friendly medical information, (5) Wish for administration-related components in a support app. The results will inform the development of a patient-centered mobile support app.

**Supplementary Information:**

The online version contains supplementary material available at 10.1007/s12687-024-00727-6.

## Introduction

Electronic Health applications such as mobile apps and web applications, can address the unique needs of cancer patients. These tools assist in diagnostics, customizing treatment plans, and education (Jongerius et al. 2019). Furthermore, eHealth solutions have been identified as empowering, improving patients’ involvement in the continuum of cancer treatment (Groen et al. 2015).

Certain cancer patients necessitate specialized care due to the hereditary nature of their condition. These patients are not only burdened by their cancer diagnosis. Additionally, they carry an increased risk to develop new independent cancers. Moreover, they can pass on the cancer predisposition to their children. Genetic factors contribute to about 5–10% of breast cancers and up to 25% of ovarian cancers (Nielsen et al. 2016). Currently, we can attribute 25% of all hereditary breast and ovarian cancers (HBOCs) to the high-risk genes *BRCA1* and *BRCA2*, while approximately 15% are associated with other HBOC risk genes such as *RAD51C*,* RAD51D*,* ATM*,* CHEK2*,* BRIP1*,* PALB2*,* BARD1*,* RECQL*,* TP53*, and *CDH1* (Easton et al. 2015). Carriers of pathogenic variants in these genes have a significantly increased risk of developing breast or ovarian cancer. These hereditary cancers often occur early in life and affect several family members (Brewer et al. 2017).

In Germany, anyone concerned about a family history of cancer can seek genetic counseling. For healthy individuals it is obligatory to undergo genetic counseling before undergoing genetic testing, as specified in the German Genetic Diagnostics Act (Human Genetic Examination Act 2009). The consultation is covered by the general as well as by private health insurance companies.

Genetic counseling prior to genetic testing considers the following content in particular: (1) Generation and discussion of the pedigree. (2) Probability of the presence of a pathogenic variant in responsible genes. (3) Risks of disease in case of identification of a pathogenic variant. (4) Benefits and harms of preventive and therapeutic options, including the option to do nothing. (5) Probability of false-negative results. (6) Significance of genetic testing for family members, including modes of inheritance.

After receiving the results of genetic diagnostics, the following contents in particular should be discussed in depth before offering preventive measures: (1) Risk of cancer development depending on genetic findings, age and concomitant diseases (natural history). (2) Mode of inheritance and probability to pass on the genetic predisposition; support to inform about options for genetic counseling and testing. (3) Options for dealing with the increased risk for cancer development, i.e. preventive options such as intensified screening, prophylactic surgery, drug therapies (4) Specific therapeutic options like PARP inhibitor or platinum-based chemotherapy. (5) Psycho-oncological support (Oncology Guidelines 2021).

This indicates that counselees are confronted with extensive and complex content. Furthermore, it is known that genetic information and interpretation are often complex and infused with misconceptions (Lanie et al. 2004). The diagnosis and treatment of cancer can often lead to cancer-related distress, as well as various mental health issues like depression and anxiety (Linden et al. 2012; Vajen et al. 2021). Additionally, being identified as a carrier of a *BRCA1* or *BRCA2* pathogenic variant can cause significant emotional distress (Voorwinden et al. 2016; Bonadona et al. 2002). No evidence of long-term adverse psychological outcomes was identified for counselees carrying a *BRCA1* or *BRCA2* pathogenic variant in long term studies (Yanes et al. 2019).

The purpose of this study was to identify barriers HBOC patients face during the genetic counseling process that may be overcome by specific digital solutions. Currently, there are two apps, ‘Digitale Gesundheitsanwendungen (DiGA),’ available in Germany that support breast cancer patients with therapy-related concerns (Register of the German Federal Institute for Drugs and Medical Devices 2023). However, there is currently no app available to support HBOC patients during the genetic counseling process in Germany. Talwar et al. analyzed the characteristics and quality of genetics and genomics mobile apps in 2018, finding that the majority of the apps had reference/resource features (95.5%), targeted health professional students (86.4%), and did not focus on specific diseases (78.5%) (Talwar et al. 2019). Only 21.6% of the apps were developed by reliable or authoritative agencies. Gasteiger et al. reached a similar conclusion when analyzing patient-facing genetic and genomic mobile apps in the UK in 2022 (Gasteiger et al. 2022). In this study, it became evident that only a few high-quality genetic patient-facing apps were available in the UK. They emphasized the need for more accessible, culturally sensitive, evidence-based apps to improve genetic literacy within patient populations and specific communities.

## Materials and methods

### Research team and reflexivity

N.A. (male) conducted a qualitative study in autumn 2022 in Hannover, Germany, to explore the needs of HBOC patients in the process of genetic counseling. N.A. is a fifth-year medical student at the University Medical Center Freiburg. He was awarded a scholarship by the Else Kröner-Fresenius Foundation to pursue a medical doctoral thesis at the Hannover Medical School. This doctoral program (DigiStrucMed) focuses on supporting projects related to the digitization of medicine. The author team, consisting of four women and four men, comprised an interdisciplinary group of researchers with diverse backgrounds. They brought together expertise from clinical genetics, biology, informatics, and medical informatics, thus contributing a wide range of experiences to their collaborative efforts. Due to valuable experience in qualitative analyses in the field of human genetics, B.V. (female, research associate at the Department of Human Genetics) provided close supervision of N.A. He had no specific prior professional or personal relations to the HBOC patients. Preliminary understandings and interpretations were discussed repeatedly with the author team. Throughout the research process, there was a constant reflection on pre-existing assumptions and personal attitudes.

### Sample

The inclusion criteria for this study were: adult patients, who received a genetic counseling at the Department of Human Genetics at Hannover Medical School, diagnosis HCOC, pathogenic variant in *BRCA1* or *BRCA2*, breast cancer and/or ovarian cancer or no cancer diagnosis. Exclusion criteria were: no written consent, no consent to be recontacted, no sufficient German language skills. For identification of potential participants B.V., N.A. and A.K.B. performed a query of our laboratory information system selected potential participants, who met the inclusion criteria and did not meet the exclusion criteria. Potential participants were recontacted by telephone by N.A. and were invited to the study. All participants provided written consent, approved by the Ethics Committee of Hannover Medical School (No. 10573_BO_K_2022). Sociodemographic variables were pseudonymized. The patients were selected purposively. All patients presented at the Department of Human Genetics for genetic counseling and testing between 2017 and 2022. Since HBOC predominantly affects women of a younger age (with an average onset age of around 45 years), our study population consisted mainly of younger women. While men with a hereditary predisposition to breast cancer can also be affected, they make up only about 1% of breast cancer cases. Hence, our study population comprised exclusively women. We offered both in-person and video interviews to all patients. All participants opted for video interviews. No patient knew the interviewer before. The interviewer introduced himself as a medical doctoral student with a particular interest in supporting patients in the process of genetic counseling by mobile support applications.

### Data collection

All patients filled out a questionnaire on their sociodemographic background (age, place of residence, educational level, occupation, migration background, siblings, children, date of diagnosis) and signed a written informed consent for the recording of the interview. The questionnaire was mailed to the participants, and upon receiving the completed and signed documents, an interview appointment was scheduled. The study was conducted according to the guidelines of the Declaration of Helsinki, and approved by the Ethics Committee of Hannover Medical School (No. 10573_BO_K_2022). For the semi-structured interviews, a predefined interview guide was used, which roughly defined the structure and topics of the interview with open questions, but still allowed sufficient flexibility for new topics. Since one of the main goals was to identify negative experiences and deficits in the genetic counseling process in order to be able to address them with the help of a support app, the first two questions addressed the experiences with the genetic counseling process.

1. Thinking back to the period from genetic counseling to shortly after (first 6 months), do you recall a specific situation in which you felt human genetic support was particularly good/helpful/positive?
2. Thinking back to the period from genetic counseling to shortly after (first 6 months), do you recall a specific situation in which you felt human genetic support was particularly bad/disappointing/negative?

Regarding the app development, the following main questions were formulated and asked if not addressed by the respondents on their own:

3. What did you think of the education about the disease/genetic predisposition?
4. What did you think of the summary letter?
5. What did you think of the education about additional support services?
6. What did you think of the psychological support?
7. Can you spontaneously think of ways in which the needs you have mentioned could be supported with the help of an app?
8. What would you have wished for? (As a general inquiry to the previously mentioned questions)

For each of these main questions, there were a number of additional questions that went into depth to obtain the most detailed information possible and were used when the interviewees did not address these aspects on their own or to generate new impulses (Döring 2023; Helfferich 2011). The interview guide represented a structure and rough direction of the topics of conversation for a more goal-oriented interview management and not a strict guideline. The development of the questions was primarily based on the authors’ extensive experience in human genetic counseling. Additionally, the current state of research was taken into account. The interview guide was checked in a pretest with non-patients (human geneticists, psychologist) for functionality and was revised. Questions were shortened or deleted and the order of the questions was changed. Preconceptions of the authors were documented prior to the survey.

Interviews were conducted by N.A. as a video call (face-to-face) at home or in the office of the Department of Human Genetics. The interviews were audio recorded and took place between the researcher and the patient only. No repeat interviews were carried out.

After each interview, an interview protocol was prepared, which contained formal information (interview number, interview duration, date) as well as information on content, such as the atmosphere of the conversation, the flow of conversation, any disruptions, topics that remained open, deviations from topics, ambiguities and the core statements of the interviewee (Döring 2023; Helfferich 2011). All data were saved on a server in the Department of Human Genetics, Hannover Medical School.

### Data analysis

The audio files of the interviews were first transcribed and digitized. Transcription was done according to predefined rules following Kuckartz (Kuckartz et al. 2022; Dresing et al. 2018). There was no paraphrasing and segregation to prevent falsification of the statements. The quotations used here were checked for meaningfulness after translation. The transcripts were not returned to patients for comments.

The interviews were evaluated according to structuring content analysis, a subform of qualitative content analysis according to Kuckartz (Kuckartz et al. 2022). For the evaluation software MAXQDA version 2022 was used. All interviews were included in the analysis.

The following steps were performed:

1. Initial text work (= systematic data exploration).
2. Development of main categories (deductive).
3. Coding data with main categories (basis coding).
4. Development of subcategories (inductive) and differentiate categories.
5. Coding data with subcategories.
6. Preparation of the results.

These steps were performed as a dynamic process and were run through several times. A deductive-inductive approach was chosen. The deductive categories were derived based on the interview guide and preliminary considerations. A pretest was conducted prior to step 3) using two randomly selected interviews to test the applicability of the main deductive categories. The category system was then improved (categories were partially deleted, redeveloped and better defined or renamed) and the material was subsequently coded using the improved category system. In the process of coding, defined coding rules were followed in order to ensure a transparent, systematic, rule-based approach. For the researchers (N.A. and B.V.) the researcher´s end point was achieved in the ninth interview, because they decided that the depth of understanding the needs of patients with HBOC was sufficient. At that timepoint, the researchers made the decision to conclude the recruitment process.

Neither the interview transcripts nor research findings were returned to patients. To ensure that the patients were not left with unaddressed unease, N.A. provided his E-mail address for further questions or comments. Some patients expressed gratitude for the conversation and indicated an interest in participating in further interviews.

The following quality criteria according to Mayring (Mayring et al. 2022) were met during the evaluation:

1. Intercoder agreement– to ensure objectivity, a second person (B.V.) was involved in the coding process. In case of ambiguity, the researchers discussed the classification of the data into the appropriate categories. The defined category system ensured an intersubjectively comprehensible analysis or category assignment.
2. Intracoder agreement was assured by N.A. through repeated coding at a later point in time.
3. Procedural documentation– the process of data collection and data analysis was documented in a way that was transparent and comprehensible to others.
4. Rule guidance– the process of data collection and data analysis was systematic and rule-governed (interview protocols, transcription rules, coding rules).
5. Proximity to the object– the data collection took place in the everyday life of the interviewees, was based on the problems of the interviewees and aimed at solving them.

## Results

### Patient characteristics

The patients have been selected based on the following criteria: All patients had HBOC (carried a pathogenic variant in *BRCA1* or *BRCA2*) and have received genetic counselling (between 2017 and 2022) at the Department of Human Genetics of the Hannover Medical School, Germany. Nine female HBOC patients participated in the study, have been interviewed and completed the interviews. Three women were invited to participate in the study but declined without giving reasons. The youngest patient was 25 years old at the time of the study, the oldest 47 years. The mean average was 38.7 years. Seven patients had a pathogenic variant in the *BRCA1* gene, two patients had a pathogenic variant in the *BRCA2* gene. Six of the nine patients had a breast cancer diagnosis. Four of them were undergoing chemotherapy at the time of genetic counseling. In the case of two patients, chemotherapy had been completed two and eight months prior to the counseling session, respectively. Three patients were not diagnosed with cancer. Supplementary Table 1 summarizes the patients’ characteristics. All participants opted for video interviews: the shortest interview lasted about 35 min, the longest about 65 min, with an average interview time of 47 min.

### Results of the qualitative analysis

Almost without exception, the interpersonal interaction between human genetics and patients was mentioned as a particularly positive experience in the process of human genetic counseling. The importance of human interaction with patients, in general, was also emphasized:

*“I have always felt that I’m in good hands at Human Genetics […]. Everyone was always very friendly. So, the human aspect was definitely very good […] The human aspect weighs much more in such a situation than the technical aspect behind it.” (Interview 3)*.

The empathy displayed by the human geneticists during the consultation and the time taken for the patients were positively mentioned.

However, patients mentioned that there are certain needs that a human geneticist may not be able to fulfill entirely.

#### Need for support in the time following genetic counseling

Most of the breast cancer patients were referred to genetic counseling to guide treatment, such as operative strategies, chemotherapy, or targeted therapy. Therefore, the patients had to manage both the recent cancer diagnosis and a possible genetic predisposition. Consequently, counseling took place in a stressful phase and the information from the counseling session could often notbe adequately absorbed:

*“When the report came, I was in the middle of therapy, bombarded with chemotherapy and, I don’t know, all kinds of other examinations and diagnoses and so on.” (Interview 2)*.

Women with a history of cancer had distinct perspectives about the content of the provided information. They desired detailed information about different treatment options. Healthy women, on the other hand, wanted detailed information about genetic mutations and inheritance. Patients, therefore, increasingly expressed the need for continued support in the form of a second counseling session sometime after the initial counseling session.

*“If you could perhaps change something*,* it might be to have a second consultation at a later time. Because […] I have just been diagnosed and then you are not so mentally receptive […]. Then you first need a bit of time to digest it*,* to think about it.” (Interview 3)*.

If such comprehensive care cannot be ensured, patients would like to receive support through an app. An app should provide information about individual consequences of their diagnosis, the next steps in the form of a “roadmap”, medical information about the treatment options, as well as information about the hereditary aspect of HBOC. It was also noted that questions emerge gradually. An app could provide guidance as further questions arise.

*“I now have children - two girls too, of course - and then of course you ask yourself: Okay, what is relevant for them? What do I have to do? When do I talk to the children? Of course, these are topics that gradually come up and that’s also something […] where I think a patient can also get counselling or support in an app like this.” (Interview 9)*.

In order to stay informed and “up-to-date” about HBOC in the future, some patients also mentioned a “news” feature about new findings, especially regarding HBOC treatments.

#### Need for support before genetic counseling

Notably, patients expressed the need for support even before the consultation, when collecting the medical reports of diseased family members and pedigree information. In particular, creating a pedigree and obtaining the necessary documents posed both an emotional and organizational challenge:

*“In between, I was on the verge of giving up everything […]. That was more or less the worst process of all, beforehand. That I had to collect everything […]. Collecting all the written documents of my family members. The phone calls […]. That was already terribly stressful beforehand […]. From the very beginning of the process, I would have liked some counseling. Some kind of help.” (Interview 1)*.

In this respect, some patients had two demands: On the one hand, emotional support to build motivation to “keep it rolling”. On the other hand, a precise explanation of why all this information is relevant and why the effort is worthwhile.

Specific suggestions for a possible support app included motivating, accompanying avatars in the form of a “female digital companion” throughout the diagnosis process, combined with push notifications as motivating reminders for the document collection process. In this context, there was also a need for a function to submit family tree documents online.

*“When you are digitally accompanied from the very beginning, by an avatar […]. That a woman in a similar situation at my age says to me: “So, you’ve decided to do it now, congratulations first of all. Congratulations, I congratulate you on deciding to get tested now!” […] “Now we’re going to do it. Together.” When a person, a virtual person, practically accompanies me on this journey from the moment I contact the genetic centre and receive the first letter about what I have to collect […]. If someone here had ever said to me: “Stay tuned! Good! Keep up the good work! It’s worth it.” (Interview 1)*.

#### Need for contact options to support services

Of all the support services, self-help groups were highlighted as particularly positive and helpful for two reasons: first, the exchange of information with fellow patients, and second, the mental support:

*“I also liked that it was directly made available to me to contact a self-help group […], that helped me a lot during therapy, because they also have a WhatsApp group and you just type in: I now have this and that, did any of you also have this? And that really, really helped a lot.” (Interview 3)*.

However, few valuable pieces of information about possible self-help groups were provided during genetic counseling sessions. Professional psychotherapy was also found to be very effective. In the case of psychological support, the difficulty of finding appointments was criticized above all.

In this respect, patients suggested providing a “contact function” with detailed contact information in the form of links to support services, such as self-help groups, psychotherapy, and social services. All patients considered a forum or chat feature for exchanging with fellow patients as a necessary function of a possible support app:

*“An app can also have a chat function, for example a forum as an exchange platform for those affected, because […] there are many breast cancer patients, but first of all to find some who also have this mutation… I think that’s also a good thing, for example, if you can […] look regionally, so to speak, so that you have like-minded people to exchange ideas with. I would find that very useful or helpful, for example.“(Interview 9)*.

#### Need for patient-friendly medical information

Interestingly, patients mentioned that the large amount of information received in the genetic counseling session was deemed useful in itself, but often not immediately relevant for the patient, thus becoming overwhelming. The density of information and comprehensibility of medical content were particularly criticized. Patients often found the counseling letter too complex and difficult to understand for laypeople. Accordingly, many patients desired simpler, more comprehensible language in the communication of medical content, as well as reduction of the content to the essentials.

*“As a medical layman, I say, it is of course very, very difficult. So, I did a lot of research on the Internet: What does that mean? What can that be?” (Interview 2)*.

An app should provide an “info board” or a “knowledge area” where patients could read about various topics depending on their own situation. These topics should include the genetic aspect of HBOC, the risk for developing cancer, genetic testing, communicating HBOC to the family and social environments, treatment and preventive measures, as well as financial aspects of HBOC.

Furthermore, patients wished for a feature such as a lexicon or glossary that would be helpful in understanding the difficult medical terminology and the complex consultation letter:

*“What you can implement well is perhaps a glossary of some kind. That you can really look up in human genetics, in the letter: What does this abbreviation mean? Or, what kind of gene description is this?“ (Interview 4)*.

#### Wish for administration-related components in a support app

Since many patients faced organizational and administrative challenges throughout the diagnosis and treatment process, there was a demand for an app feature such as a digital document management component for uploading pedigree documents, medical reports, or reimbursement applications:

*“What would also be good, of course, would be if […] you could possibly update your medical records yourself, so to speak. So perhaps if there are new diagnoses or new doctor’s letters that are relevant for human genetics, you can simply upload them via the app.” (Interview 5)*.

In this context, patients expressed the wish for a digital appointment scheduling function to schedule appointments (genetic counseling, radiology, psychology). Some patients suggested an app-integrated calendar or reminder in combination with the component for online appointments, which would be particularly helpful for prophylactic medical examination such as mammography and MRI:

*“A feature like a calendar, for example. So where you can enter your appointments, but where you can also enter your menstrual cycle somehow, because at the moment you have everything individually on your mobile phone. So that you are then reminded: Okay, well now somehow you have to make the MRI appointment, because then and then it might be convenient. So […] I would find a reminder calendar […] quite useful.” (Interview 8)*.

Additionally, patients mentioned the option to provide feedback to the developers of the app to address their individual needs:

*“I think an app like this might also need a feedback button in any case. So that you can say: Okay, we would like this and this and this.” (Interview 8)*.

## Discussion

Although all patients mentioned the interpersonal contact as an especially positive factor in the process of genetic counseling, there are needs that mobile apps could address in future. Below, we discuss the following five topics: (1) there was a need for support after genetic counseling, (2) there was a need for support before genetic counselling, (3) patients wished for contact options such as links to self-help groups and psycho-oncologists, (4) patients wished for patient-friendly information, and (5) patients suggested administration-related components for a mobile app.

### Need for support in the time following genetic counseling

The women diagnosed with breast cancer mentioned that the timing of genetic counseling was inconvenient, as it occurs during an emotionally stressful phase. Four breast cancer patients were undergoing chemotherapy at the time of genetic counseling. These women reported that the appointments for genetic counselling were also physically demanding. However, because the results of genetic testing can guide treatment, such as operative strategies, chemotherapy, and targeted therapy, it is not possible to postpone it to a later time point.

This is in line with other studies that have shown that a confirmed or suspected genetic disposition leads to further burdens, including concerns about cancer risk for children and other relatives, genetic testing, prophylactic surgery, desire to have children, and socio-legal consequences (Oncology Guidelines, 2021). Patients with HBOC are not solely concerned about their own health but also about the health of their children, grandchildren, sisters, and cousins. Feelings of guilt about passing on a predisposition are not uncommon. These fears and worries additionally hinder the cognitive absorption of all the information provided during the genetic counseling session. An app could provide specific genetic information in an easy-to-understand way, allowing patients to revisit it at a later time point determined by them.

The need for continued genetic counseling, preferably by a second consultation has also been determined by another German study: Stracke et al. interviewed women with pathogenic variants for HBOC at an academic hospital and observed information needs even several months or years after genetic counseling (Stracke et al. 2022). But the need meets a large gap in supply. Since 2009, the uptake of genetic counseling in Germany has been growing at an average rate of approximately 6% per year (Schmidtke et al. 2020) and only about 350 medical geneticists are currently available for genetic counseling for a population of 83.2 million. Furthermore, there is a lack of access to genetic support in rural areas: 88% of medical genetic outpatient clinics and practices are located in cities with more than 100,000 inhabitants and 50% in cities with more than 500,000 inhabitants (Tönnies 2016). Given the limited capacity and uneven geographical distribution of the genetic centers, the traditional support is already difficult to maintain and further genetic counseling is therefore impossible. This is not only true for Germany. The demand for genetic counseling has increased and is expected to rise increasingly in the future also in the US (Daly et al. 2021; Report of US Preventive Services Task Force 2019). One further reason for the increasing demand is the expansion of the US guidelines for genetic referral, since these now include men and women affected with a variety of cancers, regardless of age at diagnosis and family history, as well as their unaffected relatives (Hampel et al. 2015; Henneman et al. 2004). Therefore, a digital solution on demand with comprehensive and personalized information would be an alternative to counteract the need for a second genetic counseling appointment.

### Support before genetic counseling

Patients also mentioned the demand for support before the initial consultation. In particular, the process of creating a pedigree and contacting their relatives for information was perceived as very stressful. In the Department of Human Genetics as in many other institutions, patients are asked to collect pedigree information. This is necessary, since according to law, only the patients themselves are allowed to contact their family members to ask for medical details. Important details are health status, diagnoses, age of diagnoses and death. Furthermore, patients are asked for medical records of themselves and the affected family members. In line with our results, a Dutch study revealed that there are barriers for patients to share genetic information with their relatives (Henneman et al. 2004). Henneman et al. asked 1,308 participants if they were willing to share their genetic information with their own relatives. Around 10% of the participants did not want to share genetic information with their partner and children and even around 45% did not want to share this with other family members. Digital support for patients to communicate genetic information within their family, with the aim to generate a pedigree, would therefore be desirable.

### Need for contact options to support services

Some patients mentioned a need for information about self-help groups and psycho-oncological support in the interviews. This is in line with a study we conducted between 2012 and 2017 that showed that a quarter of the women requested psychological support (Vajen et al. 2021). Although counselees can be referred to an experienced psychotherapist, who is part of the team of the HBOC center, if they wish, an app could contain the compressed contact information for self-help groups and psycho-oncological support preferably as a link. Furthermore, all participants noted that a forum or chat feature would be a necessary function for a potential support app. Mobile communities built on apps can positively impact breast cancer survivors. Jung et al. have demonstrated in a randomized controlled trial with 202 patients that mobile communities can significantly reduce mental distress and increase physical activity among breast cancer survivors over a 24-week period (Jung et al. 2023).

### Need for patient-friendly medical information

Most patients mentioned that there was too much medical information, and that they did not completely understand some parts of the counseling session. It is already known that expectations about the genetic counseling session for HBOC are highly dependent on the counselee’s individual circumstances. Metcalfe et al. have observed that women with a previous diagnosis of cancer indicated that they needed more information relating to cancer treatment compared to women without cancer (Metcalfe et al. 2000). Another study has shown that women with pathogenic variants in moderate-risk genes for HBOC have special information needs compared to those with variants in high-risk genes (Stracke et al. 2022). Therefore, Henneman et al. have suggested that the process of genetic counseling should meet individual demands and should take expectations and the pre-existing lay knowledge into account (Henneman et al. 2004). An app that offers personalized information to the counselees would therefore be desirable.

Furthermore, it is well known that verbal medical information given by health support practitioners, especially genetic information, often cannot be recalled correctly. Studies on information recall have shown that up to 80% of medical information provided by health-support practitioners is forgotten and half of the information is remembered incorrectly (Kessels et al. 2003). In an app, the content could be presented in an easy and figurative language supported by illustrations. Another advantage of an app would be that access to information would always be available.

### Wish for administration-related components in a support app

Patients mentioned the wish to use the future mobile app also as a self-management tool with the possibility to organize appointments, upload medical records or to create and save their pedigree in a digital form. Notably, some patients suggested a feedback button to give direct feedback to the developers. This wish is in line with the ever-increasing desire of patients to participate in the development of patient tools. For this reason, Patient-Reported Outcome Measures (PROMs) and Patient-Reported Experience Measures (PREMs) were developed and are already in use to evaluate telemedicine applications (Knapp et al. 2021).

### Limitations of the study

Although this study provides valuable insights into experiences and needs of women with HBOC, who presented for genetic counseling and testing at the Department of Human Genetics at the Hanover Medical School, the limitations of the study need to be considered. Our project was performed in a single center with a limited number of participants. This study did not include patients with ovarian cancer or other HBOC-related cancers. The study also did not include carriers of other HBOC genes. These are significant limitations as perspectives of these additional HBOC patients and therefore stakeholders in a future app were not elicited. Furthermore, the participants gave their retrospective assessment. Although we offered in-person interviews to all patients, they all preferred video interviews. The reasons cited were related to organizational circumstances, such as difficulties in appointment scheduling and the inconvenience of a long journey. It has already been shown that one of the disadvantages of video interviews is technical barriers (Lobe et al. 2022). However, all patients felt comfortable with the technical aspects. Furthermore, it is known that less-structured interviews can pose challenges for encouraging participation in video interviews (Clarke and Braun 2013). In our study, this was not the case due to the utilization of a semi-structured interview guide and the highly relevant topic for the participants. However, recent years of qualitative research has shown that video interviews can lead to a reduction in code density (Lobe et al. 2022).

### Practice implications

In this study, nine women provided us with detailed insights into the needs of a subset of patients—both healthy women and those with a history of breast cancer—who have a *BRCA1* or *BRCA2* mutation, within the context of the genetic counseling process.These insights may be beneficial for genetic counselors, gynecologists, patient support groups, patients themselves, and for the further development of mobile support apps for HBOC patients. Participatory design should be followed during the development process to ensure responsiveness to evolving needs. Integrating feedback tools could ensure the recording of patients` needs (Vaisson et al. 2021). Patient-Reported Outcome Measures (PROMs) and Patient-Reported Experience Measures (PREMs), already used to evaluate telemedicine applications, could be suitable measurement tools (Knapp et al. 2021). The utilization of eHealth solutions, such as support apps in medical care, could help address the significant gap between supply and demand. Evaluation of the two already available DIGAs (certified medical products in Germany) for patients with breast cancer has shown that the use of these apps/internet-based interventions led to a significant reduction in psychological distress, an antifatigue efficacy as well as an increase in physical activity and quality of life (Wolff et al. 2023; Holtdirk et al. 2021). Thus, these solutions may offer direct benefits to patients, recognized as tools for patient education and for enhancing patient engagement in the treatment process.

## Conclusion

Although patients highlighted the empathic approach of the human geneticists in this study, some barriers were mentioned that make the use of a digital mobile support, such as an app for HBOC patients, worthwhile. The app should aim to enhance support by delivering personalized and easily understandable information tailored to the specific needs of each patient. Importantly, it should operate flexibly, allowing users to access support and information at their convenience. Furthermore, the application should play a proactive role, extending its assistance to patients both before and after genetic counseling sessions. In doing so, a support app could effectively address identified barriers and elevate the overall quality and accessibility of support for HBOC patients throughout their healthcare journey. Additionally, our study suggests that patients and patient-support groups should be invited to and involved in the fundamental stages of creating innovative app-based interventions, which aligns with the general recommendation of using participatory design when developing new patient tools.

## Electronic supplementary material

Below is the link to the electronic supplementary material.

Supplementary Material 1

## Data Availability

The data presented in this study are available on request from the corresponding author.
