# Supplementary material for: Deciphering the needs of patients with hereditary breast and ovarian Cancer in the Process of Genetic Counseling to Inform the Development of a Mobile Support App: a qualitative study in Germany
Source: J Community Genet. 2024 Aug 19;15(6):603–13. doi: 10.1007/s12687-024-00727-6 (PMC11645348; doi:10.1007/s12687-024-00727-6)
Supplement: Supplementary file 1 — Supplementary Material 1 [file 12687_2024_727_MOESM1_ESM.docx]

**Supplementary material**

**Deciphering the needs of patients with Hereditary Breast and Ovarian Cancer in the process of genetic counseling to inform the development of a mobile support app: a qualitative study in Germany**

Nils Ammon^1^, Chiara Reichert^2^, Thomas Kupka^2^*,* Steffen Oeltze-Jafra^2^*,* Anke Katharina Bergmann^1^*,* Brigitte Schlegelberger^1^, Dominik Wolff^2^, Beate Vajen^1 *^

**Supplementary Table 1**: Sociodemographic data of the interviewed patients

| **Patient characteristics** | | | |
| --- | --- | --- | --- |
| ***characteristics*** | ***mean years (range)*** | ***n (%)*** | ***mean number per patient (range)*** |
| **Age** | 38.7 (25-47) |  |  |
| **Medical history** |  |  |  |
| Healthy women |  | 3 (33.3) |  |
| Women with breast cancer |  | 6 (67.7) |  |
| Age at first diagnosis of breast cancer | 35.5 (24-42) |  |  |
| Time genetic counseling until interview | 3 (0.67-5.9) |  |  |
| **Pathogenic variant in** |  |  |  |
| *BRCA1* |  | 7 (78.8) |  |
| *BRCA2* |  | 2 (22.2) |  |
| **Family situation** |  |  |  |
| Having children |  | 5 (55.6) |  |
| Number of children |  |  | 0.67 (0-2) |
| Having siblings |  | 9 (100) |  |
| Number of siblings |  |  | 1.78 (1-4) |
| **Migration background** |  | 3 (33.4) |  |
| **Highest level of education** |  |  |  |
| High school graduate |  | 2 (22.2) |  |
| University degree |  | 7 (78.8) |  |
| **Residence** |  |  |  |
| >100.000 |  | 2 (22.2) |  |
| 20.000-100.000 |  | 2 (22.2) |  |
| <20.000 |  | 5 (55.6) |  |

Note. N = 9.
